# Impact of mechanical stimulation on the chondrogenic processes in human bone marrow aspirates modified to overexpress *sox9* via rAAV vectors

**DOI:** 10.1186/s40634-017-0097-1

**Published:** 2017-06-21

**Authors:** Jagadeesh K. Venkatesan, Janina Frisch, Ana Rey-Rico, Gertrud Schmitt, Henning Madry, Magali Cucchiarini

**Affiliations:** 1 Center of Experimental Orthopaedics, Saarland University Medical Center, Kirrbergerstr. Bldg 37, D-66421 Homburg/Saar, Germany; 2 Department of Orthopaedic Surgery, Saarland University Medical Center, Kirrbergerstr. Bldg 37, D-66421 Homburg/Saar, Germany

**Keywords:** Cartilage repair, Bone marrow aspirates, Recombinant adeno-associated virus, Chondrogenic differentiation, Mechanical stimulation

## Abstract

**Background:**

Evaluation of gene-based approaches to target human bone marrow aspirates in conditions of mechanical stimulation that aim at reproducing the natural joint environment may allow to develop improved treatments for articular cartilage injuries. In the present study, we investigated the potential of rAAV-mediated *sox9* gene transfer to enhance the chondrogenic differentiation processes in human bone marrow aspirates under established hydrodynamic conditions compared with the more commonly employed static culture conditions.

**Methods:**

Fresh human bone marrow aspirates were transduced with rAAV-FLAG-h*sox9* (40 μl) and maintained for up to 28 days in chondrogenic medium under mechanically-induced conditions in dynamic flow rotating bioreactors that permit tissue growth and matrix deposition relative to static culture conditions. The samples were then processed to examine the potential effects of *sox9* overexpression on the cellular activities (matrix synthesis, proliferation) and on the chondrogenic differentiation potency compared with control treatments (absence of rAAV vector; reporter rAAV-*lacZ*, rAAV-RFP, and rAAV-*luc* gene transfer).

**Results:**

Prolonged, significant *sox9* overexpression via rAAV was achieved in the aspirates for at least 28 days when applying the rAAV-FLAG-h*sox9* construct, leading to higher, prolonged levels of matrix biosynthesis and to enhanced chondrogenic activities relative to control treatments especially when maintaining the samples under mechanical stimulation. Administration of *sox9* however did not impact the indices of proliferation in the aspirates. Remarkably, *sox9* gene transfer also durably delayed hypertrophic and osteogenic differentiation in the samples regardless of the conditions of culture applied *versus* control treatments.

**Conclusions:**

The current observations show the value of genetically modifying human bone marrow aspirates upon mechanical stimulation by rAAV *sox9* as a promising strategy for future treatments to improve cartilage repair by implantation in lesions where the tissue is submitted to natural mechanical forces.

## Background

The articular cartilage is a highly specialized tissue that provides a smooth, low friction weightbearing in diarthrodial joints while protecting bones from damaging loads. The absence of vascularity and lymphatic drainage in the cartilage restricts the ability of this tissue for self-repair in response to injury like after trauma or during degenerative osteoarthritis (Buckwalter & Mankin 1998). While various options are available in the clinics including autologous chondrocyte transplantation and marrow-stimulating procedures, none of them promote the full reproduction of the original hyaline cartilage surface in patients (proteoglycans, type-II collagen) that can durably withstand mechanical load (Cucchiarini et al. 2014). New, promising avenues of clinical investigation to address such challenges are based on the direct, convenient administration of bone marrow aspirates that carry chondrogenically competent mesenchymal stem cells (MSCs) to sites of articular cartilage lesions (Kim et al. 2014; Slynarski et al. 2006) rather than of isolated MSCs that necessitate more complex steps of preparation and expansion (Cucchiarini et al. 2014). However, the outcomes of such clinically adapted treatments revealed again the production of a firbocartilaginous repair tissue in the lesions that is of lesser structural and mechanical quality than the original hyaline cartilage organization.

Therapeutic gene-based modification of bone marrow aspirates prior to re-implantation in sites of injury may enhance the chondroreparative processes to promote the formation of an improved cartilage repair tissue (Cucchiarini et al. 2014; Frisch & Cucchiarini 2016). Among the different factors reported for their chondrogenic properties (transforming growth factor beta - TGF-β, bone morphogenetic proteins - BMPs, basic fibroblast growth factor - FGF-2, insulin-like growth factor I - IGF-I, zinc finger protein 145 - ZNF145, Indian hedgehog - Ihh, cartilage oligomeric matrix protein - COMP) (Cucchiarini et al. 2011; Frisch et al. 2015a; Frisch et al. 2016a; Haleem-Smith et al. 2012; Ivkovic et al. 2010; Kawamura et al. 2005; Liu et al. 2011; Neumann et al. 2013; Pagnotto et al. 2007; Steinert et al. 2012), the sex-determining region Y-type high-mobility group box 9 (SOX9) transcription factor might be the best suited candidate to achieve this goal. Remarkably, this transcription factor promotes both chondrocyte differentiation and cartilage formation (Bi et al. 1999) while advantageously restricting terminal differentiation and hypertrophy (Akiyama et al. 2004) and displays superior reparative activities in cartilage lesions *in vivo* over TGF-β and IGF-I (Frisch et al. 2016b). Interestingly, *sox9* gene transfer has been already performed to initiate chondrogenic pathways in isolated human MSCs via nonviral (Babister et al. 2008) and adenoviral vectors (Kupcsik et al. 2010), but the use of such vectors for human gene therapy purposes remains limited by their relatively low and/or short-term efficacy and potential immunogenicity (Frisch et al. 2015b). In contrast, recombinant adeno-associated viral (rAAV) vectors that allow to achieve higher and prolonged levels of transgene expression may be more adapted for translational approaches (Frisch et al. 2015b). In this regard, we provided evidence of the capability of this vector class to target isolated human bone marrow-derived MSCs (Venkatesan et al. 2012) and most notably of human bone marrow aspirates (Rey-Rico et al. 2015a) maintained in static conditions. As chondrogenic processes in the bone marrow *in vivo* are subjected to mechanical forces (O’Conor et al. 2013; Terraciano et al. 2007), the goal of the present study was thus to further investigate the potential chondrogenic effects mediated by *sox9* gene transfer via rAAV in human bone marrow aspirates upon continuous mechanical stimulation, using dynamic conditions that aim at reproducing a more natural environment (Madry et al. 2013).

The present findings demonstrate that effective, prolonged overexpression of *sox9* using rAAV vectors stimulates the chondrogenic differentiation processes in human bone marrow aspirates compared with control treatments, most particularly when applying mechanical forces, with advantageously reduced premature hypertrophy and terminal differentiation, further supporting the concept of providing such a construct to treat articular cartilage defects in patients.

## Methods

### Chemicals and reagents

All reagents were from Sigma (Munich, Germany) unless otherwise indicated. Recombinant TGF-β was from R&D Systems (Wiesbaden, Germany). The anti-SOX9 (C-20) antibody was from Santa Cruz Biotechnology (Heidelberg, Germany), the anti-type-II collagen (II-II6B3) antibody from the NIH Hybridoma Bank (University of Iowa, Ames, USA), and the anti-type-X collagen (COL-10) antibody from Sigma. Biotinylated secondary antibodies and ABC were from Vector Laboratories (Grünberg, Germany). Luciferase activity was determined with the Luciferase Assay System (Promega GmbH, Mannheim, Germany) and normalized to total cellular proteins using the BCA protein assay kit (Pierce Thermo Scientific, Fisher Scientific GmbH, Schwerte, Germany). The Cell Proliferation Reagent WST-1 was from Roche Applied Science (Mannheim, Germany). The type-II collagen contents were measured with the native type-II collagen Arthrogen-CIA Capture ELISA kit (Chondrex, Redmond, WA, USA) and those for type-X collagen using a COL-10 ELISA (Antibodies-Online, Aachen, Germany).

### Human bone marrow aspirates

Bone marrow aspirates (~15 ml; 0.4–1.4 x 10^9^ cells/ml) were obtained from the distal femur of patients undergoing total knee arthroplasty (*n* = 28, age 73 ± 5 years) (Frisch et al. 2015a; Rey-Rico et al. 2015a). The study was approved by the Ethics Committee of the Saarland Physicians Council (*Ärztekammer des Saarlandes*, application with reference number Ha06/08). All patients provided informed consent to participate in the study before inclusion in the study. All procedures were in accordance with the Helsinki Declaration. All patients provided informed consent to report individual patient data. Aliquots containing MSCs (Frisch et al. 2015a; Ivkovic et al. 2010; Rey-Rico et al. 2015a) were placed in 96-well plates (150 μl aspirate/well) and maintained in DMEM with 100 U/ml penicillin and 100 μl/ml streptomycin (pen-strep) (basal medium) and 10% FBS (growth medium) at 37 °C in a humidified atmosphere with 5% CO_2_ for subsequent analyses.

### Plasmids and rAAV vectors

The constructs were derived from pSSV9, an AAV-2 genomic clone (Samulski et al. 1987; Samulski et al. 1989). rAAV-*lacZ* carries the *lacZ* gene for *E. coli* β-galactosidase (β-gal), rAAV-RFP the *Discosoma* sp. red fluorescent protein (RFP) gene, rAAV-*luc* the Firefly luciferase (*luc*) gene, and rAAV-FLAG-h*sox9* a 1.7-kb FLAG-tagged human SOX9 sequence, all under the control of the cytomegalovirus immediate-early (CMV-IE) promoter (Cucchiarini et al. 2011; Frisch et al. 2015a; Rey-Rico et al. 2015a; Venkatesan et al. 2012). The vectors were packaged as conventional (not self-complementary) vectors using a helper-free, two-plasmid transfection system in the 293 packaging cell line (an adenovirus-transformed human embryonic kidney cell line) with the packaging plasmid pXX2 and the Adenovirus helper plasmid pXX6 as previously described (Rey-Rico et al. 2015a). The vector preparations were purified by dialysis and titered by real-time PCR (Cucchiarini et al. 2011; Frisch et al. 2015a; Rey-Rico et al. 2015a; Venkatesan et al. 2012), averaging 10^10^ transgene copies/ml (viral particles-to-functional vectors = 500/1) (Beck et al. 1999).

### rAAV-mediated gene transfer

Aliquoted aspirates were transduced for 90 min with the rAAV vectors (40 μl each vector, i.e. 8 x 10^5^ functional recombinant viral particles, multiplicity of infection - MOI = 10) or let untreated as previously described (Frisch et al. 2015a; Rey-Rico et al. 2015a). A mixture of fibrinogen (17 mg/ml)/thrombin (5 U/ml) (Baxter, Volketstwil, Switzerland) was then added to the aspirates (70 μl per aspirate) and the samples were evenly distributed in a patient-matched manner in 1.5-ml Eppendorf tubes (one sample per tube, 200 μl medium) (static cultures) and in dynamic flow rotating bioreactors (RCCV-110; Synthecon, Houston, TX) (mechanically-stimulated cultures) that permit tissue growth and matrix deposition under optimal hydrodynamic conditions for chondrogenesis relative to static culture (Madry et al. 2013) for up to 28 days (Madry et al. 2013) using defined chondrogenic medium of DMEM high glucose (4.5 g/l), pen-strep, ITS^+^ Premix (insulin 6.25 μg/ml, transferring 6.25 μg/ml, selenous acid 6.25 μg/ml, linoleic acid 5.35 μg/ml, and bovine serum albumin 1.25 μg/ml), pyruvate (1 mM), ascorbate 2-phosphate (37.5 μg/ml), with dexamethasone (10^-7^ M) and TGF-β (1 ng/ml) 24 h after application of rAAV (Frisch et al. 2015a; Rey-Rico et al. 2015a).

### Transgene expression

Transgene expression was determined by detection of live fluorescence, X-Gal staining, analysis of luciferase activity (Luciferase Assay System) with normalization to total cellular proteins, or by immunohistochemical analyses (SOX9) on histological sections using specific primary antibodies, biotinylated secondary antibodies, and the ABC method with diaminobenzidine (DAB) as the chromogen (Cucchiarini et al. 2011; Frisch et al. 2015a; Rey-Rico et al. 2015a; Venkatesan et al. 2012). To control for secondary immunoglobulins, samples were processed with omission of the primary antibody. Samples were examined directly by light microscopy (Olympus BX 45; Hamburg, Germany) or by fluorescent microscopy using an Olympus microscope with a 568-nm filter (CKX41).

### Histological and immunohistochemical analyses

The aspirates were collected, fixed in 4% buffered formalin, and dehydrated in graded alcohols for paraffin embedding (Frisch et al. 2015a; Rey-Rico et al. 2015a; Venkatesan et al. 2012). Paraffin-embedded sections (5 μm) were stained with hematoxylin eosin (H&E) (cellularity), safranin O (matrix proteoglycans) and alizarin red (matrix mineralization) according to routine protocols (Cucchiarini et al. 2011). Fast green was used as a counterstain for the evaluations of transduction efficiencies. Expression of type-II and -X collagen was detected by immunohistochemistry using specific antibodies (Cucchiarini et al. 2011; Frisch et al. 2015a; Rey-Rico et al. 2015a; Venkatesan et al. 2012). To control for secondary immunoglobulins, samples were processed with omission of the primary antibody. Samples were examined by light microscopy (Olympus BX 45).

### Histomorphometric analyses

The wet weights, the perimeters, the transduction efficiencies (ratio of X-Gal-stained cells to the total number of cells), the % of SOX9-stained cells, the cell densities (cells/mm^2^), and the histological and immunohistochemical grading scores (safranin O, alizarin red, type-II and -X collagen) were measured at three standardized sites using replicate samples and ten serial sections per condition using SIS AnalySIS (Olympus), Adobe Photoshop (Adobe Systems, Unterschleissheim, Germany), and Scion Image (Scion Corporation, Frederick, MD, USA) (Cucchiarini et al. 2011; Frisch et al. 2015a; Rey-Rico et al. 2015a; Venkatesan et al. 2012). Safranin O and alizarin red staining and type-II and -X collagen immunostaining were scored for uniformity and intensity according to a modified Bern Score grading system (Rey-Rico et al. 2015b) as: 0 (no staining), 1 (heterogeneous and/or weak staining), 2 (homogeneous and/or moderate staining), 3 (homogeneous and/or intense staining), and 4 (very intense staining). Sections were scored blind by two individuals with regard to the conditions.

### Biochemical assays

Cell proliferation in the samples was assessed using the Cell Proliferation Reagent WST-1 with OD^450 nm^ proportional to the cell numbers according to the manufacturer’s recommendations and as previously described (Cucchiarini et al. 2011; Venkatesan et al. 2012). Aspirates were digested in papain solution using previously described protocols (Cucchiarini et al. 2011; Frisch et al. 2015a; Venkatesan et al. 2012). The DNA contents were determined using Hoechst 33258, the proteoglycan contents by binding to the DMMB dye, and those for type-II and -X collagen by ELISA (Cucchiarini et al. 2011; Frisch et al. 2015a; Venkatesan et al. 2012) and data were normalized to total cellular proteins. All measurements were performed with a GENios spectrophotometer/fluorometer (Tecan, Crailsheim, Germany).

### Total RNA extraction and real-time RT-PCR analyses

Total cellular RNA was extracted from the cultures using the RNeasy Protect Mini Kit with an on-column RNase-free DNase treatment (Qiagen, Hilden, Germany) (Cucchiarini et al. 2011; Frisch et al. 2015a; Venkatesan et al. 2012). RNA was eluted in 30 μl RNase-free water. Reverse transcription was carried out with 8 μl of eluate using the 1^st^ Strand cDNA Synthesis kit for RT-PCR (AMV) (Roche Applied Science). Repeated preliminary quantitative evaluations revealed reliable amounts of material in the samples. An aliquot of the cDNA product (2 μl) was amplified by real-time PCR using the Brilliant® SYBR® Green QPCR Master Mix (Stratagene, Agilent Technologies, Waldbronn, Germany) (Cucchiarini et al. 2011; Frisch et al. 2015a; Venkatesan et al. 2012) on a Mx3000P® QPCR operator system (Stratagene) as follows: (95 °C, 10 min), amplification by 40 cycles (denaturation at 95 °C, 30 s; annealing at 55 °C, 1 min; extension at 72 °C, 30 s), denaturation (95 °C, 1 min), and final incubation (55 °C, 30 s). The primers (Invitrogen GmbH) used were: SOX9 (chondrogenic marker) (forward 5′-ACACACAGCTCACTCGACCTTG-3′; reverse 5′-GGGAATTCTGGTTGGTCCTCT-3′), type-II collagen (COL2A1) (chondrogenic marker) (forward 5′-GGACTTTTCTCCCCTCTCT-3′; reverse 5′-GACCCGAAGGTCTTACAGGA-3′), type-X collagen (COL10A1) (marker of hypertrophy) (forward 5′-CCCTCTTGTTAGTGCCAACC-3′; reverse 5′-AGATTCCAGTCCTTGGGTCA-3′), alkaline phosphatase (ALP) (osteogenic marker) (forward 5′-TGGAGCTTCAGAAGCTCAACACCA-3′; reverse 5′-ATCTCGTTGTCTGAGTACCAGTCC-3′), matrix metalloproteinase 13 (MMP13) (marker of terminal differentiation) (forward 5′-AATTTTCACTTTTGGCAATGA-3′; reverse 5′-CAAATAATTTATGAAAAAGGGATGC-3′), runt-related transcription factor 2 (RUNX2) (osteogenic marker) (forward 5′-GCAGTTCCCAAGCATTTCAT-3′; reverse 5′-CACTCTGGCTTTGGGAAGAG-3′), β-catenin (mediator of the Wnt signaling pathway for osteoblast lineage differentiation) (forward 5′- CAAGTGGGTGGTATAGAGG-3′; reverse 5′-GCGGGACAAAGGGCAAGA-3′), and glyceraldehyde-3-phosphate dehydrogenase (GAPDH) (housekeeping gene and internal control) (forward 5′-GAAGGTGAAGGTCGGAGTC-3′; reverse 5′-GAAGATGGTGATGGGATTTC-3′) (all 150 nM final concentration) (Cucchiarini et al. 2011; Frisch et al. 2015a; Venkatesan et al. 2012). Control conditions included reactions using water and non-reverse-transcribed mRNA. Specificity of the products was confirmed by melting curve analysis and agarose gel electrophoresis. The threshold cycle (Ct) value for each gene of interest was measured for each amplified sample using the MxPro QPCR software (Stratagene) and values were normalized to GAPDH expression using the 2^-ΔΔCt^ method as previously employed (Cucchiarini et al. 2011; Frisch et al. 2015a; Venkatesan et al. 2012).

### Statistical analysis

Each condition was performed in triplicate in three independent experiments. All samples were tested for all the experiments. Data are expressed as mean ± standard deviation (SD) of separate experiments. The t-test and Mann-Whitney Rank Sum Test were employed where appropriate. *P* values of less than 0.05 were considered statistically significant.

## Results

### Maintenance of human bone marrow aspirates in dynamic and static culture conditions upon rAAV-mediated gene transfer

We first examined whether freshly aspirated bone marrow from human donors can be maintained over time in an environment favorable to the formation of a three-dimensional composition. To achieve this goal, aspirates were embedded in a fibrin gel and monitored for their stability in dynamic and static culture conditions over a period of 28 days (Madry et al. 2013).

**Fig. 1:**
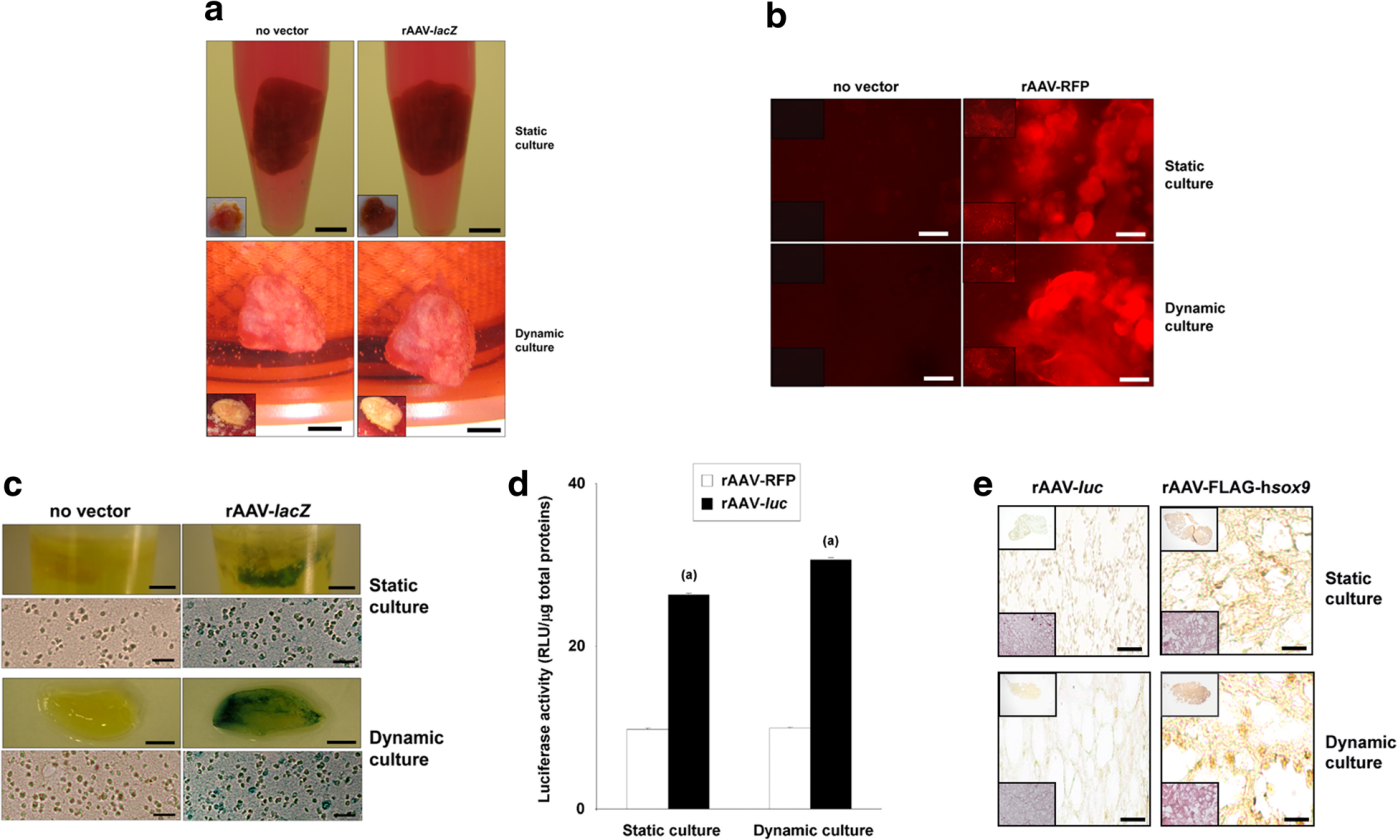
Detection of transgene expression in rAAV-transduced human bone marrow aspirates in static *versus* dynamic culture conditions. Aspirates (150 μl) were transduced with rAAV-RFP, rAAV-*lacZ*, rAAV-*luc*, or rAAV-FLAG-h*sox9* (40 μl each vector) or let untransduced for maintenance in dynamic or static culture conditions for up to 28 days (*n* = 4 independent samples tested in triplicate per vector treatment and culture condition in three independent experiments) as described in the Methods. **a** Macroscopic views of the aspirates are presented for each cultivation systems (day 21, scale bars: 1.5 cm for the static cultures and 2 cm for the dynamic cultures; insets: day 28), **b** Detection of RFP expression by live fluorescence (day 21, scale bars: 50 μm; top insets: day 7; bottom insets: day 28), **c** detection of *lacZ* expression (day 21) by X-Gal staining (macroscopic views, scale bars: 1.5 cm for the static cultures and 2 cm for the dynamic cultures) and following histological processing of the samples (magnification x20, scale bars: 100 μm, all representative data), **d** detection of *luc* expression by analysis of luciferase activity with normalization to total cellular proteins, and **e** detection of SOX9 (day 28, magnification x40, scale bars: 40 μm; top insets: complete section, magnification x4; bottom insets: H&E staining, magnification x40; all representative data) as described in the Methods. ^a^Statistically significant relative to rAAV-RFP application

A macroscopic view of aspirates that received no vector treatment revealed that both types of cultivation systems allowed for the formation of a compact structure of the samples, without significant difference in the perimeters of the samples (2.7 ± 0.3 *versus* 3.4 ± 0.1 mm and 3.0 ± 0.3 *versus* 3.6 ± 0.2 mm in dynamic and static conditions after 21 and 28 days, respectively, *P* ≥ 0.420; *P* ≥ 0.283 between similar conditions when comparing days 21 and 28) (Fig. 1a). There was also no significant difference in the wet weight between the samples (0.28 ± 0.01 *versus* 0.24 ± 0.02 g and 0.30 ± 0.02 *versus* 0.27 ± 0.02 g in dynamic and static conditions after 21 and 28 days, respectively, *P* ≥ 0.063 and *P* ≥ 0.102 between similar conditions when comparing days 21 and 28). When rAAV-*lacZ* was provided to the aspirates, no noticeable modifications of the overall appearance of the aspirates were observed between systems (perimeters: 2.7 ± 0.2 *versus* 3.2 ± 0.1 mm and 3.1 ± 0.2 *versus* 3.3 ± 0.2 mm in dynamic and static conditions after 21 and 28 days, respectively, *P* ≥ 0.157 and *P* ≥ 0.650 between similar conditions when comparing days 21 and 28; wet weights: 0.28 ± 0.01 *versus* 0.23 ± 0.02 g and 0.29 ± 0.02 *versus* 0.25 ± 0.02 g in dynamic and static conditions after 21 and 28 days, respectively, *P* ≥ 0.072 and *P* ≥ 0.371 between similar conditions when comparing days 21 and 28) or when comparing conditions with or without vector treatment (perimeters: *P* ≥ 0.527; wet weights: *P* ≥ 0.293) (Fig. 1a).

### Efficient rAAV-mediated transgene expression in human bone marrow aspirates maintained in dynamic and static culture conditions

We next assessed whether successful genetic modification of the aspirates could be achieved over time in dynamic and static culture conditions upon rAAV-mediated gene transfer.

Sustained, efficient RFP expression was noted in rAAV-RFP-transduced aspirates both in dynamic and static culture conditions already after 7 days and for at least 28 days (the longest time point examined) (Madry et al. 2013) compared with control transduction (absence of vector treatment), showing no visible differences between the two cultivation systems (Fig. 1b). In good agreement, prolonged, effective *lacZ* expression was detected in the aspirates upon administration of rAAV-*lacZ* both in dynamic and static conditions *versus* control treatment (absence of vector treatment) (Fig. 1c), with transduction efficiencies reaching ~ 80–85% in either cultivation system (*versus* below 2% in the controls, *P* ≤ 0.001) as seen on histological sections from aspirates (Fig. 1c). Significant, elevated levels of luciferase activity were also achieved over time in rAAV-*luc*-transduced aspirates compared with control (rAAV-RFP) treatment in the two culture conditions (30.6 ± 0.1 *versus* 9.9 ± 0.1 and 26.3 ± 0.2 *versus* 9.8 ± 0.1 RLU/μg total proteins in dynamic environment and static system on day 21, respectively, i.e. an up to 3.1-fold difference, *P* ≤ 0.001) (Fig. 1d). Prolonged, significant expression of the candidate *sox9* sequence was also noted in the aspirates following administration of rAAV-FLAG-h*sox9* compared with control (rAAV-*luc*) transduction (85% *versus* 6% and 84% *versus* 3% SOX9-stained cells in dynamic environment and static system on day 28, respectively, i.e. an up to 28-fold difference, *P* ≤ 0.001) (Fig. 1e).

### Effects of rAAV-mediated gene transfer upon the metabolic activities in human bone marrow aspirates maintained in dynamic and static culture conditions

We then tested whether gene transfer via rAAV altered the proliferative, matrix biosynthetic, and chondrogenic activities in bone marrow aspirates maintained over time in dynamic and static culture conditions while evaluating the possible effects of candidate *sox9* gene transfer on these processes using this vector class.

Maintenance of the aspirates in dynamic culture conditions led to significant increases in the cell densities in the samples compared with static culture, regardless of the presence or absence of vector (rAAV-*lacZ*, rAAV-*luc*, rAAV-FLAG-h*sox9*) treatment and of the time point evaluated (up to 1.7-fold difference, *P* ≤ 0.001) (Fig. 1c and d; Tables 1 and 2), showing no deleterious effects of rAAV in either system tested relative to each respective control (untreated) condition (*P* ≥ 0.342). Administration of the *sox9* vector did not modify the levels of cell proliferation in the aspirates in any of the systems tested compared with the control treatments at any time point examined (*P* ≥ 0.151). These findings were corroborated by an estimation of the proliferation rates in the aspirates using a WST-1 assay, showing increased proliferative activities in dynamic *versus* static culture conditions at any time point examined, with or without vector (up to 3.3-fold difference, *P* ≤ 0.001) (Tables 1 and 2), without detrimental effect of rAAV (*P* ≥ 0.898) while again *sox9* treatment did not influence the outcomes (*P* ≥ 0.555). Finally, similar results were obtained when monitoring the DNA contents in the aspirates (up to 1.2-fold increase in dynamic *versus* static culture conditions independently of the presence or absence of vector treatment and at any time point examined, *P* ≤ 0.001; no deleterious effect of rAAV: *P* ≥ 0.648; no effect of *sox9* application: *P* ≥ 0.342) (Tables 1 and 2).

**Table 1:** Biochemical assays in the rAAV-transduced human bone marrow aspirates (day 21)

Assay	Static culture			Dynamic culture		
	no vector	*luc*	*sox9*	no vector	*luc*	*sox9*
Cell densities (cells/mm^2^)	4556 (94)	5453 (87)	5641 (95)	7044 (104)^c^	9101 (112)^c^	9157 (108)^c^
WST-1 (OD^450 nm^)	0.320 (0.031)	0.351 (0.046)	0.402 (0.048)	1.037 (0.118)^c^	1.002 (0.107)^c^	1.056 (0.122)^c^
DNA (μg/mg total proteins)	0.31 (0.12)	0.32 (0.17)	0.35 (0.17)	0.36 (0.14)^c^	0.37 (0.17)^c^	0.42 (0.16)^c^
Proteoglycans (mg/mg total proteins)	0.006 (0.002)	0.007 (0.003)	0.009 (0.005)^a,b^	0.009 (0.002)^c^	0.010 (0.003)^c^	0.016 (0.004)^a,b,c^
Proteoglycans (mg/μg DNA)	0.019 (0.001)	0.022 (0.002)	0.026 (0.003)^a,b^	0.025 (0.001)^c^	0.027 (0.002)^c^	0.038 (0.002)^a,b,c^
Type-II collagen (μg/mg total proteins)	0.018 (0.002)	0.019 (0.001)	0.024 (0.001)^a,b^	0.021 (0.001)^c^	0.022 (0.002)^c^	0.040 (0.003)^a,b,c^
Type-II collagen (μg/μg DNA)	0.058 (0.003)	0.059 (0.002)	0.069 (0.003)^a,b^	0.065 (0.002)^c^	0.068 (0.003)^c^	0.095 (0.004)^a,b,c^
Type-X collagen (μg/mg total proteins)	0.022 (0.004)	0.024 (0.007)	0.012 (0.006)^a,b^	0.020 (0.003)	0.021 (0.005)	0.011 (0.002)^a,b^
Type-X collagen (μg/μg DNA)	0.071 (0.003)	0.075 (0.004)	0.034 (0.004)^a,b^	0.068 (0.002)	0.070 (0.004)	0.026 (0.002)^a,b^

Values are given as mean (SD) with *n* = 4 independent samples tested in triplicate per vector treatment and culture condition in three independent experiments. Statistically significant relative to ^a^no vector treatment, ^b^rAAV-*luc* application, and ^c^static culture

**Table 2:** Biochemical assays in the rAAV-transduced human bone marrow aspirates (day 28)

Assay	Static culture			Dynamic culture		
	no vector	*luc*	*sox9*	no vector	*luc*	*sox9*
Cell densities (cells/mm^2^)	4389 (88)	4987 (91)	5084 (72)	6796 (98)^c^	7046 (103)^c^	7892 (76)^c^
WST-1 (OD^450 nm^)	0.298 (0.024)	0.304 (0.032)	0.328 (0.037)	0.787 (0.095)^c^	0.881 (0.088)^c^	0.904 (0.063)^c^
DNA (μg/mg total proteins)	0.29 (0.08)	0.30 (0.11)	0.34 (0.09)	0.35 (0.12)^c^	0.32 (0.14)^c^	0.38 (0.09)^c^
Proteoglycans (mg/mg total proteins)	0.009 (0.002)	0.010 (0.002)	0.012 (0.004)^a,b^	0.011 (0.002)^c^	0.012 (0.003)^c^	0.017 (0.003)^a,b,c^
Proteoglycans (mg/μg DNA)	0.031 (0.003)	0.033 (0.003)	0.037 (0.002)^a,b^	0.034 (0.002)^c^	0.037 (0.002)^c^	0.045 (0.004)^a,b,c^
Type-II collagen (μg/mg total proteins)	0.016 (0.002)	0.018 (0.002)	0.023 (0.001)^a,b^	0.020 (0.001)^c^	0.021 (0.002)^c^	0.039 (0.002)^a,b,c^
Type-II collagen (μg/μg DNA)	0.055 (0.002)	0.058 (0.003)	0.067 (0.003)^a,b^	0.062 (0.002)^c^	0.065 (0.002)^c^	0.102 (0.003)^a,b,c^
Type-X collagen (μg/mg total proteins)	0.021 (0.003)	0.022 (0.004)	0.014 (0.003)^a,b^	0.019 (0.002)	0.020 (0.003)	0.010 (0.002)^a,b^
Type-X collagen (μg/μg DNA)	0.072 (0.002)	0.073 (0.003)	0.041 (0.003)^a,b^	0.069 (0.003)	0.071 (0.002)	0.026 (0.002)^a,b^

Values are given as mean (SD) with *n* = 4 independent samples tested in triplicate per vector treatment and culture condition in three independent experiments. Statistically significant relative to ^a^no vector treatment, ^b^rAAV-*luc* application, and ^c^static

**Fig. 2:**
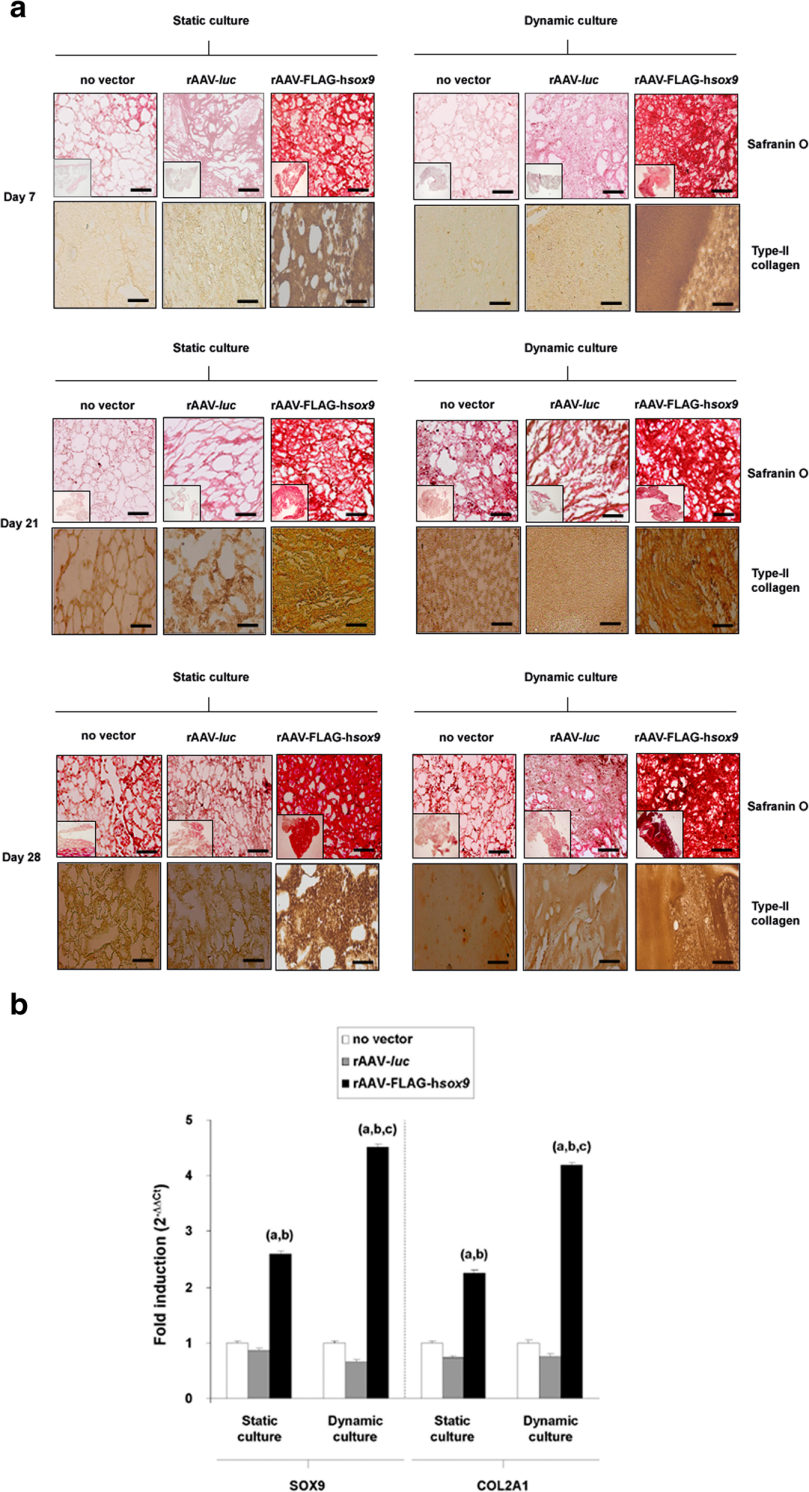
Evalution of cartilage-specific components in rAAV-transduced human bone marrow aspirates in static *versus* dynamic culture conditions. Aspirates (150 μl) were prepared and transduced with rAAV-*luc* or rAAV-FLAG-h*sox9* as described in Fig. 1 or let untransduced for maintenance in dynamic or static culture conditions for up to 28 days (*n* = 4 independent samples tested in triplicate per vector treatment and culture condition in three independent experiments) as described in the Methods. The samples were processed (**a**) for safranin O staining and to detect the expression of type-II collagen after 7, 21, and 28 days as described in the Methods (magnification x10, scale bars: 200 μm; insets: complete section, magnification x4; all representative data) and (**b**) to evaluate the gene expression profiles (SOX9 and COL2A1, with GAPDH serving as a housekeeping gene and internal control) after 21 days by real-time RT-PCR amplification as described in the Methods. Ct values were obtained for each target and GAPDH as a control for normalization, and fold inductions (relative to untreated aggregates) were measured by using the 2^-ΔΔCt^ method. Statistically significant relative to ^a^no vector treatment, ^b^rAAV-*luc* application, and ^c^static culture

Enhanced levels of cartilage-specific matrix biosynthesis was also achieved in the samples in dynamic compared with static culture, already starting after 7 days of culture in the presence of rAAV-FLAG-h*sox9* and for the whole period of evaluation (28 days) and after 21 days in control cultures (no vector treatment, rAAV-*luc* transduction), as noted on histological sections from aspirates processed of effective chondrogenesis (Madry et al. 2013) for safranin O staining and type-II collagen immunostaining (Fig. 2a) and scored to grade the staining intensities (Rey-Rico et al. 2015b) (up to 1.3- and 5.7-fold difference for safranin O and type-II collagen, respectively, *P* ≤ 0.038) (Table 3). No detrimental effect of rAAV was observed in any of the systems examined at any time point examined (*P* ≥ 0.196). Notably, application of the *sox9* vector increased the levels of matrix synthesis in the aspirates in both systems at any time point examined compared with the control treatments (up to 8- and 14-fold difference for safranin O and type-II collagen, respectively, *P* ≤ 0.001). In good agreement, durable increases in the proteoglycan and type-II collagen contents were noted in dynamic *versus* static culture conditions at any time point examined, with or without vector (up to 1.8- and 1.7-fold difference for the proteoglycan and type-II collagen contents, respectively, *P* ≤ 0.001) (Tables 1 and 2). There was no deleterious effect of rAAV (*P* ≥ 0.195) and again a durable stimulating effect of *sox9* compared with the control treatments (up to 1.8- and 1.9-fold difference for the proteoglycan and type-II collagen contents, respectively, *P* ≤ 0.001).

**Table 3:** Histomorphometric analyses in the rAAV-transduced human bone marrow aspirates (day 21)

Assay	Static culture			Dynamic culture		
	no vector	*luc*	*sox9*	no vector	*luc*	*sox9*
Safranin O staining	0.3 (0.2)	0.5 (0.2)	2.4 (0.3)^a,b^	1.8 (0.3)^c^	1.9 (0.3)^c^	3.1 (0.2)^a,b,c^
Type-II collagen immunostaining	2.1 (0.1)	2.2 (0.2)	3.2 (0.1)^a,b^	2.7 (0.2)^c^	2.6 (0.2)^c^	3.8 (0.2)^a,b,c^
Alizarin red staining	3.3 (0.3)	3.1 (0.4)	0.8 (0.4)^a,b^	3.2 (0.1)	3.2 (0.3)	0.7 (0.2)^a,b^
Type-X collagen immunostaining	3.2 (0.2)	3.0 (0.1)	0.7 (0.2)^a,b^	3.1 (0.4)	3.0 (0.2)	0.8 (0.1)^a,b^

Values are given as mean (SD) with *n* = 4 independent samples tested in triplicate per vector treatment and culture condition in three independent experiments. Safranin O and alizarin red staining and type-II and -X collagen immunostaining were scored as described in Table 5 (Rey-Rico et al. 2015b). Statistically significant relative to ^a^no vector treatment, ^b^rAAV-*luc* application, and ^c^static culture

An analysis of the gene expression profiles performed by real-time RT-PCR after 21 days of effective chondrogenesis (Madry et al. 2013) revealed higher levels of SOX9 and COL2A1 expression in the presence of *sox9* relative to the control treatments (up to 6.7- and 2.9-fold difference for SOX9 in dynamic and static culture, respectively, and up to 5.6- and 3.1-fold difference for COL2A1 in dynamic and static culture, respectively, *P* ≤ 0.001). The effect that was even more marked in dynamic compared with static culture conditions (up to 1.7-difference for SOX9 and 1.8-fold difference for COL2A1 with *sox9*, respectively, *P* ≤ 0.001) (Fig. 2b).

### Effects of rAAV-mediated gene transfer upon the hypertrophic and terminal differentiation processes in human bone marrow aspirates maintained in dynamic and static culture conditions

We finally monitored potential deleterious effects of rAAV gene transfer on the hypertrophic and terminal differentiation in bone marrow aspirates maintained over time in dynamic and static culture conditions while testing the influence of the candidate *sox9* gene transfer on such processes using this vector class.

**Fig. 3:**
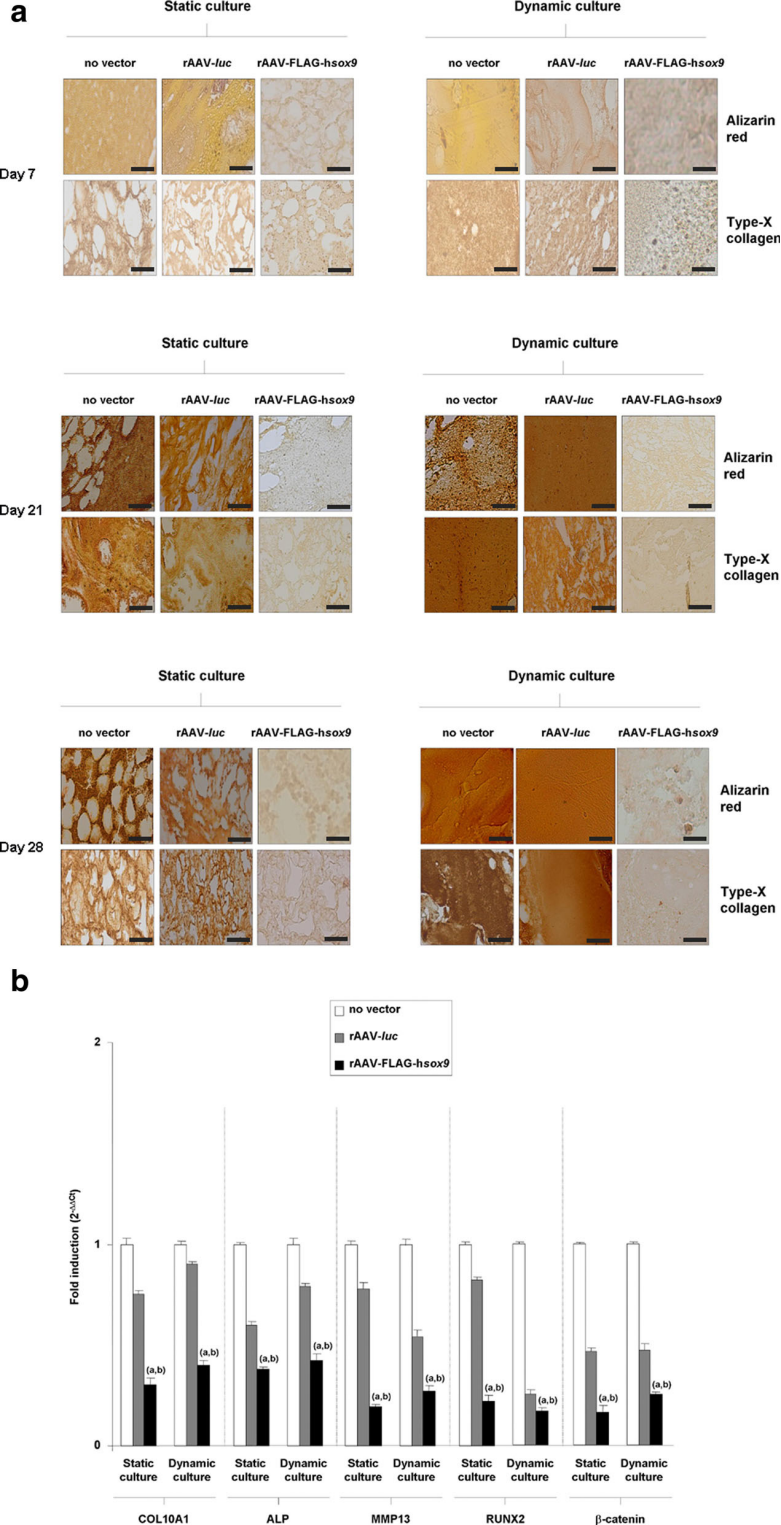
Evaluation of hypertrophic and terminal differentiation processes in rAAV-transduced human bone marrow aspirates in static *versus* dynamic culture conditions. Aspirates (150 μl) were prepared and transduced with rAAV-*luc* or rAAV-FLAG-h*sox9* as described in Fig. 1 and 2 or let untransduced for maintenance in dynamic or static culture conditions for up to 28 days (*n* = 4 independent samples tested in triplicate per vector treatment and culture condition in three independent experiments) as described in the Methods. The samples were processed (**a**) for alizarin *red* staining and to detect the expression of type-X collagen after 7, 21, and 28 days as described in the Methods (magnification x10, scale bars: 200 μm, all representative data) and (**b**) to evaluate the gene expression profiles (COL10A1, ALP, MMP13, RUNX2, and β-catenin, with GAPDH serving as a housekeeping gene and internal control) after 21 days by real-time RT-PCR amplification as described in the Methods and in Fig. 2. Ct values were obtained for each target and GAPDH as a control for normalization, and fold inductions (relative to untreated aggregates) were measured by using the 2^-ΔΔCt^ method. Statistically significant relative to ^a^no vector treatment and ^b^rAAV-*luc* application

Remarkably, administration of the *sox9* vector significantly and durably decreased the levels of matrix mineralization and of hypertrophic/terminal differentiation in the aspirates both in dynamic and static culture conditions compared with the control treatments (no vector, rAAV-*luc*) as noted on histological sections from aspirates processed after 21 and 28 days for alizarin red staining and type-X collagen immunostaining, respectively (Fig. 3a). Scores grading the staining intensities (Rey-Rico et al. 2015b) revealed up to 10- and 7.7-fold difference for alizarin red and type-X collagen, respectively) (*P* ≤ 0.012) (Tables 3 and 4). As expected, hypertrophy and terminal differentiation was not documented early on (day 7) (Fig. 3a and Table 5). No detrimental effect of rAAV was noted in any of the systems examined at any time point examined (*P* ≥ 0.187) and no difference was reported between dynamic and static culture conditions (*P* ≥ 0.187). These findings were supported by an analysis of the type-X collagen contents in the aspirates, with lower amounts in the presence of *sox9 versus* control treatments at any time point examined (up to 2.7-fold difference, *P* ≤ 0.001) (Tables 1 and 2) and without effect of the vectors in either system tested nor of the culture conditions (*P* ≥ 0.187). An analysis of the gene expression profiles performed by real-time RT-PCR after 21 days of effective chondrogenesis (Madry et al. 2013) revealed lower levels of COL10A1 expression in the presence of *sox9* relative to the control treatments (up to 2.5- and 3.3-fold difference in dynamic and static culture, respectively, *P* ≤ 0.001) but again without significant difference between dynamic and static culture conditions (*P* ≥ 0.141) (Fig. 3b). This was accompanied by decreases in the expression profiles of ALP (up to 2.2- and 2.6-fold difference in dynamic and static culture, respectively), MMP13 (up to 3.7- and 5.3-fold difference in dynamic and static culture, respectively), RUNX2 (up to 5.9- and 4.3-fold difference in dynamic and static culture, respectively), and β-catenin (up to 4- and 6.3-fold difference in dynamic and static culture, respectively) upon rAAV-FLAG-h*sox9* administration compared with the control treatments (always *P* ≤ 0.001). Again, there was no significant difference between dynamic and static culture conditions (*P* ≥ 0.121) (Fig. 3b).

**Table 4:** Histomorphometric analyses in the rAAV-transduced human bone marrow aspirates (day 28)

Assay	Static culture			Dynamic culture		
	no vector	*luc*	*sox9*	no vector	*luc*	*sox9*
Safranin O staining	1.8 (0.2)	1.8 (0.3)	3.3 (0.2)^a,b^	1.8 (0.2)	2.0 (0.2)	4.0 (0.1)^a,b,c^
Type-II collagen immunostaining	2.2 (0.2)	2.2 (0.1)	3.1 (0.2)^a,b^	2.4 (0.2)	2.5 (0.1)	3.6 (0.2)^a,b,c^
Alizarin red staining	3.2 (0.2)	3.0 (0.3)	0.9 (0.3)^a,b^	3.5 (0.1)	3.4 (0.2)	0.7 (0.1)^a,b^
Type-X collagen immunostaining	3.0 (0.2)	3.1 (0.1)	0.8 (0.2)^a,b^	3.0 (0.2)	3.1 (0.2)	0.6 (0.1)^a,b^

Values are given as mean (SD) with *n* = 4 independent samples tested in triplicate per vector treatment and culture condition in three independent experiments. Safranin O and alizarin red staining and type-II and -X collagen immunostaining were scored as described in Table 5 (Rey-Rico et al. 2015b). Statistically significant relative to ^a^no vector treatment, ^b^rAAV-*luc* application, and ^c^static culture

**Table 5:** Histomorphometric analyses in the rAAV-transduced human bone marrow aspirates (day 7)

Assay	Static culture			Dynamic culture		
	no vector	*luc*	*sox9*	no vector	*luc*	*sox9*
Safranin O staining	0.3 (0.2)	0.3 (0.1)	2.0 (0.2)^a,b^	0.2 (0.1)	0.3 (0.1)	2.6 (0.3)^a,b,c^
Type-II collagen immunostaining	0.2 (0.1)	0.3 (0.1)	2.8 (0.3)^a,b^	0.4 (0.1)	0.4 (0.2)	3.3 (0.2)^a,b,c^
Alizarin red staining	0.2 (0.1)	0.2 (0.2)	0.1 (0.1)	0.3 (0.2)	01. (0.1)	0.2 (0.1)
Type-X collagen immunostaining	0.1 (0.1)	0.1 (0.1)	0.2 (0.1)	0.2 (0.1)	0.2 (0.1)	0.1 (0.1)

Values are given as mean (SD) with *n* = 4 independent samples tested in triplicate per vector treatment and culture condition in three independent experiments. Safranin O and alizarin red staining and type-II and -X collagen immunostaining were scored for uniformity and intensity according to a modified Bern Score grading system (Rey-Rico et al. 2015b) as: 0 (no staining), 1 (heterogeneous and/or weak staining), 2 (homogeneous and/or moderate staining), 3 (homogeneous and/or intense staining), and 4 (very intense staining). Statistically significant relative to ^a^no vector treatment, ^b^rAAV-*luc* application, and ^c^static culture

## Discussion

Reproducing an original, biomechanically functional tissue in sites of cartilage damage remains one of the most challenging issue in the clinics as both spontaneous cartilage repair and currently available guided procedures, including those based on the single-step administration of chondrogenically competent bone marrow aspirates (Kim et al. 2014; Slynarski et al. 2006), lead to the formation of a poorly organized tissue of lesser mechanical quality than the hyaline cartilage. In this regard, gene transfer strategies may provide effective tools to enhance the chondroreparative processes in such samples especially when subjected to mechanical stimulation as a means to reproduce the natural environment of the joint. We thus examined the potential benefits of delivering the highly chondrogenic transcription factor *sox9* to human bone marrow aspirates using the clinically adapted rAAV vectors upon continuous maintenance in dynamic culture conditions (flow rotating bioreactors) that permit tissue growth and matrix deposition under optimal hydrodynamic conditions for chondrogenesis relative to static culture (Madry et al. 2013) to extend our previous findings when samples were kept in a static environment (Rey-Rico et al. 2015a).

The present findings first reveal that gene transfer via rAAV allows for a highly effective and durable expression of several reporter genes (RFP, *lacZ*, *luc*) and of the candidate *sox9* sequence in human bone marrow aspirates in a mechanically active environment over an extended period of time (at least 28 days), with transduction efficiencies in the range of those noted when applying static culture conditions (80–85%) (Rey-Rico et al. 2015a). Sustained levels of *sox9* expression using rAAV led to prolonged increases in the production of chondrogenic markers and of cartilage-specific matrix components (SOX9, proteoglycans, type-II collagen) in the aspirates compared with control treatments (at least 28 days), especially upon mechanical stimulation that may exert direct effects on cell function by hydrodynamic forces and/or indirect flow-induced modifications in mass transfer of nutrients and metabolites. This is in good agreement with the properties of the transcription factor (Bi et al. 1999) and with our preliminary observations in static cultures (Rey-Rico et al. 2015a) or when providing the same construct to isolated human bone marrow-derived MSCs (Venkatesan et al. 2012). In contrast, administration of rAAV *sox9* did not modulate the proliferative processes in the samples over time *versus* control treatments in any of the systems evaluated (even though mechanical stimulation led to increased activities), again consistent with the known activities of SOX9 (Akiyama et al. 2004) and with our findings in isolated human bone marrow-derived MSCs (Venkatesan et al. 2012).

Interestingly, modification of the aspirates via rAAV *sox9* promoted a durable, advantageous reduction of matrix mineralization and hypertrophic/terminal differentiation profiles relative to control treatments, although no difference was noted between samples undergoing mechanical stimulation or kept in static culture conditions. This was probably due to decreased expression levels of the osteogenic transcription factor RUNX2 that controls COL10A1, ALP, and MMP13 expression (Enomoto et al. 2000; Frisch et al. *in press*) and of the β-catenin signaling mediator that regulates osteoblast lineage differentiation via *sox9* gene transfer and overexpression (Akiyama et al. 2004; Topol et al. 2009; Yamashita et al. 2009). These results are concordant with the reported effects of SOX9 on terminal differentiation and calcification and with our preliminary observations in static culture (Rey-Rico et al. 2015a) and in isolated human bone marrow-derived MSCs (Venkatesan et al. 2012).

For comparison, Kupcsik *et al*. (Kupcsik et al. 2010) reported that *sox9* gene transfer in isolated human bone marrow-derived MSCs enhanced the production of matrix components under mechanical stimulation but not when modified cells were maintained in static culture conditions. However, these authors employed a different approach than that tested here, using less effective, highly immunogenic adenoviral vectors that are not adapted for clinical applications to treat non-lethal disorders such as those affecting the articular cartilage and at much higher MOI (100 instead of 10 here with rAAV, i.e. a 10-fold difference) in conditions of cell seeding in polyurethane scaffolds for maintenance under superimposed compression in a custom-made bioreactor over a shorter period of chondrogenic evaluation (14 instead of 28 days). In addition, the use of bone marrow aspirates here represent a much more convenient, single-step approach and advance relative to the use of MSCs that need to be isolated, thoroughly characterized, and expanded in culture prior to reimplantation in the recipient. Also, our scaffold-free approach is less complex than that employed by these authors, again allowing for direct, simple strategies for future *in vivo* applications. Besides, rAAV might be better suited for translation into the clinics as they appear to be much less immunogenic and more effective over time than vectors based on adenoviruses (Frisch et al. 2015b).

## Conclusion

Overall, the results of the present study using conditions of mechanical stimulation extend our previous work when aspirates were maintained in static culture (Rey-Rico et al. 2015a) and further support the idea of using the current candidate rAAV *sox9* vector for administration in sites of cartilage injury in patients as a means to improve the chondrogenerative processes in a tissue that is naturally submitted to mechanical forces in the joint. Work is ongoing to test whether the therapeutic effects noted *in vitro* here, including those on hypertrophy and terminal differentiation, may also occur in relevant, orthotopic experimental models *in vivo* with native mechanical environment (Madry et al. 2013). To achieve this goal, genetically modified bone marrow aspirates may be provided by implantation in focal cartilage lesions to observe the formation of an improved cartilage tissue in a native (cellular, biochemical) environment, a study on itself that also necessitates to translate the current work in human samples in an evaluation of animal bone marrow concentrates.

## Abbreviations

ALP: Alkaline phosphatase
AMV: Avian myeloblastosis virus
BMP: Bone morphogenetic protein
cDNA: Complementary deoxyribonucleic acid
CMV-IE: Cytomegalovirus immediate-early
COL10A1: Type-X collagen
COL2A1: Type-II collagen
COMP: Cartilage oligomeric matrix protein
Ct: Threshold cycle
DAB: Diaminobenzidine
DMEM: Dulbecco’s modified Eagle’s medium
DMMB: Dimethylmethylene blue
DNA: Deoxyribonucleic acid
DNase: Deoxyribonuclease
ELISA: Enzyme-linked immunosorbent assay
FBS: Fetal bovine serum
FGF-2: Basic fibroblast growth factor
GAPDH: Glyceraldehyde-3-phosphate dehydrogenase
IGF-I: Insulin-like growth factor I
Ihh: Indian hedgehog
luc: Firefly luciferase
MMP: Matrix metalloproteinase
MOI: Multiplicity of infection
mRNA: Messenger ribonucleic acid
MSCs: Mesenchymal stem cells
OD: Optical density
QPCR: Quantitative polymerase chain reaction
rAAV: Recombinant adeno-associated virus
RFP: Red fluorescent protein
RNA: Ribonucleic acid
RNase: Ribonuclease
RT-PCR: Reverse-transcriptase polymerase chain reaction
RUNX2: Runt-related transcription factor 2
SD: Standard deviation
SOX: Sex-determining region Y-type high mobility group box
TGF-β: Transformation growth factor beta
Wnt: Wingless/Int
ZNF145: Zinc finger protein 145
β-gal: β-galactosidase
